# Genetic Map Construction and Quantitative Trait Locus (QTL) Detection of Six Economic Traits Using an F_2_ Population of the Hybrid from *Saccharina longissima* and *Saccharina japonica*

**DOI:** 10.1371/journal.pone.0128588

**Published:** 2015-05-26

**Authors:** Jing Zhang, Tao Liu, Rongfang Feng, Cui Liu, Shan Chi

**Affiliations:** 1 Qilu University of Technology, Jinan, Shandong, People’s Republic of China; 2 College of Marine Life Sciences, Ocean University of China, Qingdao, Shandong, People’s Republic of China; 3 Guangdong Institute of Microbiology, Guangzhou, Guangdong, People’s Republic of China; Pennsylvania State University, UNITED STATES

## Abstract

*Saccharina* (*Laminaria*) is one of the most important economic seaweeds. Previously, four genetic linkage maps of *Saccharina* have been constructed and five QTLs have been identified. However, they were not enough for its breeding. In this work, *Saccharina longissima* (♀) and *Saccharina japonica* (♂), which showed obvious differences in morphology and genetics, were applied in hybridization to yield the F_2_ mapping population with 102 individuals. Using these 102 F_2_ hybrids, the genetic linkage map of *Saccharina* was constructed by MapMaker software based on 37 amplified fragment length polymorphisms (AFLPs), 22 sequence-related amplified polymorphisms (SRAPs) and 139 simple sequence repeats (SSRs) markers. Meanwhile, QTL analysis was performed for six economic traits. The linkage map constructed in this research consisted of 422 marker loci (137 AFLPs, 57 SRAPs and 228 SSRs), which formed 45 linkage groups (LGs) with an average marker space of 7.92 cM; they spanned a total length of 2233.1 cM, covering the whole estimated genome size. A total of 29 QTLs were identified for six economic traits, which explained 1.06 to 64.00% of phenotypic variation, including three QTLs for frond length (FL) and raw weight (RW), five QTLs for frond width (FW), two QTLs for frond fascia width (FFW) and frond thickness (FT), and fourteen QTLs for base shape (BS). The results of this research will improve the breeding efficiency and be beneficial for marker-assisted selection (MAS) schemes in *Saccharina* breeding.

## Introduction

As one of the most important economic seaweed in China, *Saccharina* has been used as food and raw industrial materials for algin, mannitol and iodine extraction [1]. Meanwhile, it plays a key role in maintaining offshore ecological balance [2]. In recent years, increased market demands for dry food of *Saccharina* required both higher productivity and better economic traits. Thus, prominent variety breeding is quite crucial. So far, more than ten high-yield varieties of *Saccharina* have been bred and cultivated widely [3–7], which has made remarkable progress in *Saccharina* cultivation industry in China. However, some problems still exist in *Saccharina* breeding. For example, current breeding strategy depends on direct phenotype selection and multi-generation inbreeding which is time-consuming and labor-intensive. Furthermore, many traits related to economic production and quality are quantitatively inherited and determined by the combined interaction between genetic and environmental factors [8]. This will decrease the accuracy and efficiency of conventional breeding depending on direct phenotype selection. Therefore, understanding the relationship between variations in DNA sequences and variations in phenotypes for these quantitative traits will accelerate the selective breeding.

Genetic linkage map is an essential tool for plant molecular breeding because it has the properties of neutrality, lack epistasis, and is simply Mendelian inherited [9]. Owing to genetic linkage, DNA markers can be used to detect the presence of allelic variation in genes underlying these traits [10]. They could offer great scope for improving the efficiency by carrying out selection not on phenotype directly but on genotype [11]. Moreover, in recent years, quantitative trait loci (QTLs) analysis has become a key tool for identifying the markers linked to the objective traits, facilitating estimation of the minimum number of genomic regions that affect a trait [12].

Genetic linkage map and QTLs analysis have been successfully applied to important agricultural plants [13–15] and recently to aquaculture species such as Atlantic salmon, rainbow trout, tilapiaand and so on [16]. There were four genetic linkage maps of *Saccharina* till now [17–20] mainly using dominant AFLP markers and only a few co-dominant SSR markers. The lack of co-dominant markers was the common weakness of previous *Saccharina* genetic linkage maps. In addition, the first QTL mapping has only recently become available and only five QTLs associated with two economic traits (frond length and frond width) have been identified based on linkage map [19]. In this research, a high-density genetic linkage map was constructed with AFLP, SRAP as well as SSR markers using F_2_ progenies of *S*. *longissima* and *S*. *japonica* parents, and 29 QTLs were identified related to six economic traits including frond length, frond width, frond fascia width, frond thickness, raw weight and base shape. Compared with previous genetic linkage maps of *Saccharina*, we increased the marker number, especially more co-dominant markers, in order to construct a genetic map with higher density. In addition, QTLs associated with six traits which related to economic production and quality were identified for the first time. These results will be helpful to the genetic improvement and provide a new strategy for *Saccharina* breeding.

## Materials and Methods

### Mapping population construction

*S*. *longissima* female gametophyte clones and *S*. *japonica* male gametophyte clones were selected as parents in the interspecific hybridization to yield the F_1_ generation. The parental gametophyte clones used for hybridization were haploid and provided by the Culture Collection of Seaweed at the Ocean University of China. 102 F_2_ progenies, derived from F_1_ self-crossing, were used as a segregating population for genetic linkage map construction. *S*. *longissima* strains had long and narrow frond, while *S*. *japonica* had short and wide frond. Obvious morphological and genetic differences could result in a higher proportion of polymorphic loci in the hybrid progeny which was beneficial to the construction of high-density genetic linkage map [21] and detecting the QTLs related to traits. Isolation of gametophyte clones and crossing of gametophytes used the method described by Zhang et al. [7].

### Traits Examination

**Fig 1:**
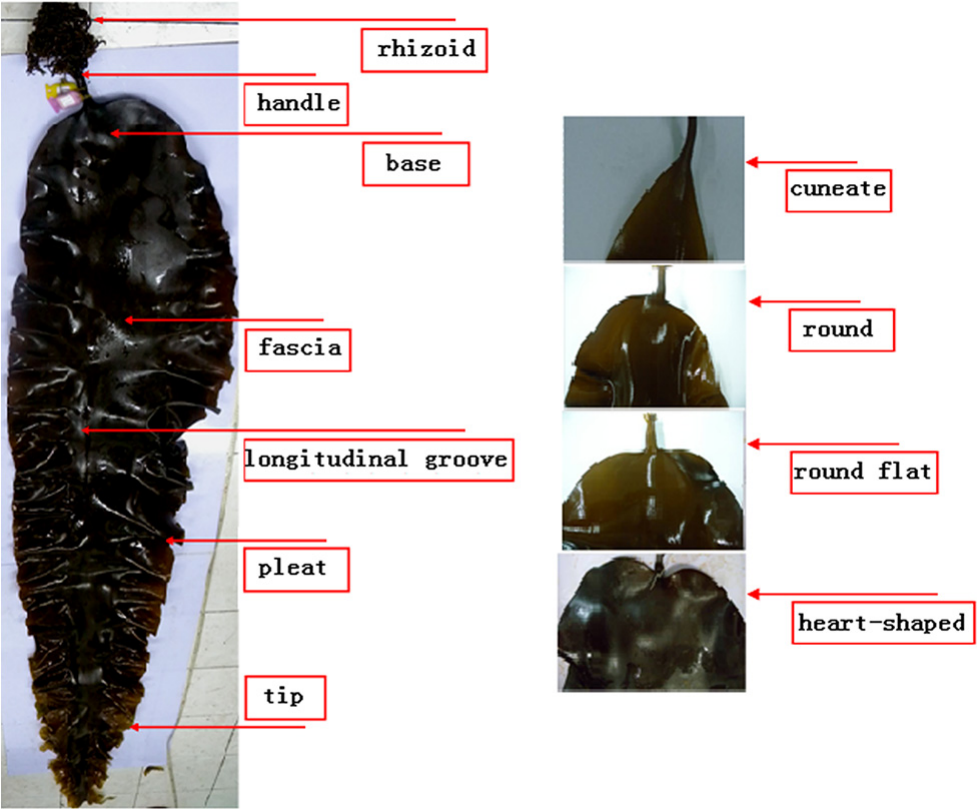
Phenotypic traits of *Saccharina* and four types (cuneate, round, round flat, and heart-shaped) of base shape.

Six economic traits were detected in this study, including frond length (FL), frond width (FW), frond fascia width (FFW), frond thickness (FT), raw weight (RW) and base shape (BS). FL was measured from the joint of the frond and stipe to the tip of the frond. FW was the width of the widest part of frond, while FFW was at the widest part of the frond between the two longitudinal groove. FT was the average thickness of three points that divided the frond into four fragments of equal length. Average individual RW was calculated by dividing the total weight of two ropes by the total number of individuals counted each time. BS was divided into four types, such as cuneate, round, round flat, and heart-shaped which were assigned 1.00, 2.00, 3.00, 4.00 respectively. Phenotypic traits of *Saccharina* and four types (cuneate, round, round flat, and heart-shaped) of base shape were shown in Fig 1. SPSS 17.0 software was applied for the correlations coefficient analysis between the traits and the normal distribution analysis of each trait.

### AFLP, SRAP and SSR analysis

Genomic DNA of F_2_ progenies and the parents were isolated with improved CTAB method [22].

AFLP analysis was based on the method described by Vos et al. [23]. Eight EcoRI primers and eight MseI primers were synthesized at Invitrogen, Shanghai, China and used to form 64 primer combinations. These primer combinations that generated clear, highly polymorphic, and reproducible DNA bands between two parents were selected and used in subsequent experiments.

SRAP was a novel molecular marker technique aimed at amplifying open reading frames (ORFs) [24]. Primers synthesized at Invitrogen, Shanghai, China with clearly separated bands, stable amplification and rich polymorphism were selected from 88 primer combinations which were reported by Ding et al. [25]. SRAP analysis was performed according to the procedure described by Li and Quiros with minor modification [24].

There have been few SSR markers of *Saccharina* reported until now. 60 SSR primers from Billot et al. [26], Liu et al. [27], Shi et al. [28] and Wang et al. [29] were selected in this study firstly. Then together with 266 our newly developed SSR primers from the (AC)_n_ enriched genomic library [30], a total of 326 primers were synthesized at Invitrogen, Shanghai, China. The PCR was performed under the following conditions: 5 min at 95°C, then 35 cycles of 1 min at 92°C, 1 min at appropriate annealing temperature, and 1 min at 72°C, and a final extension step of 10 min at 72°C. These SSR primer combinations were selected and used in genetic linkage mapping, which generated polymorphic and reproducible DNA bands between two parents as well as generated objective bands from more than two F_2_ individuals at the same time.

All the PCR products were separated on 6% denaturing polyacrylamide gel and visualized by silver staining. A 20 bp DNA ladder was used as a reference marker for allele size determination.

AFLP and SRAP marker loci were scored as dominant markers, and all polymorphic markers present in one parent and absent in the other one were scored. At the same time, the segregation at each marker locus was checked for deviation from Mendelian segregation ratio (3:1) by a Chi-square goodness-of-fit test. All dominant markers in this research were named according to the types of primers used to generate them and also their size. For example, marker A64f147 indicated that AFLP marker was analyzed with a size of 147 bp and marker e1m2f138 indicated that SRAP marker was analyzed with a size of 138 bp. SSR markers were scored as co-dominant markers and markers that were segregated in a 1:2:1 Mendelian segregation ratio in the F_2_ mapping population were used to construct genetic linkage map. SSR markers were renamed according to information of the source and the size of the microsatellite bands. For instance, marker S253f157 indicated that SSR marker from (AC)_n_ enriched genomic library was analyzed with a size of 157 bp; marker E12f134 referred to 134-bp fragment generated by EST-SSR marker from Wang et al. [29]. In addition, marker H152f181 and G61f331 indicated that SSR markers from the previous reports of *Saccharina* were analyzed. Genotyping data were assembled in a unique file (.raw) to elaborate the genetic linkage map.

### Map construction and QTL mapping

A Chi-squared test was used to assess Mendelian segregation distortion of all polymorphic loci data in F_2_ population before linkage analysis.

MapMaker (EXP 3.0) was used to build the genetic linkage map [31]. A minimum LOD score of 3.0 was first used to associate loci into initial linkage groups. Then, the LOD score was reduced to bridge some intervals. To determine the correct marker order within the linkage groups, multi-point analysis was performed using ‘order’, ‘try’ and ‘map’ commands for groups containing more than five loci and using ‘comp’ for groups containing less than or equal to five loci.

The convert from recombination frequency to map distance was done by Kosambi function [32], and genome size was estimated using methods described by Postlethwait et al. [33]. The graphic representations of linkage groups were drawn by MapChart 2.2 software [34].

QTL detection was performed on the software package Windows QTL Cartographer 2.5 according to the user manual [35]. Composite interval mapping (CIM) method was introduced to detect QTLs and the estimation of effects. CIM was one method of QTL mapping by combining interval mapping with multiple regression. The basis of CIM which by combining interval mapping with multiple regression was an interval test in which the test statistic on a marker interval was made to be unaffected by QTLs located outside a defined interval [36]. The genome-wide significant threshold for each trait was determined by carrying out 1,000 permutations at P<0.01. A QTL position was determined at the local maximum of the LOD plot curve in the region under consideration. The proportion of phenotypic variance explained by a single QTL was calculated as the square of the partial correlation coefficient. Gene action type of QTL was determined according to the criterion suggested by Stuber et al. [37]. QTL nomenclature referred to McCouch et al. [38]. Four parts were included in the QTLs: q + FL/FW/FFW/FT/RW/BS (frond length, frond width, frond fascia width, frond thickness, raw weight and base ship) + a number (the serial number of the linkage groups) + a number (the serial number of the QTL controlling a trait).

## Results

### Phenotypic traits analysis

The two parent strains have obvious differences in the frond length and width. The frond length of female parent had an average of 573.00 cm, which was significantly longer than 228.91cm of male parent (P = 1.30E^-18^<0.01). While the frond width of female parent had an average of 25.00 cm, which was significantly narrower than 29.96 cm of male parent (P = 0.0005<0.01). The average frond length (395.09 cm) of the F_1_ family was close to the average value of the parents. The frond width of F_1_ had an average of 31.57 cm, showing advantage over the parents. Basic data of the two traits (frond length and frond width) in parent lines and F_1_ family were shown in Table 1.

**Table 1:** Basic data of the two traits (frond length and frond width) in parent lines and F_1_ family.

Trait	*S*. *longissima* (Female parent)		*S*. *japonica* (Male parent)		F_1_	
	Mean	SD	Mean	SD	Mean	SD
FL (cm)	573.00	89.92	228.91	66.92	395.09	44.08
FW(cm)	25.00	4.90	29.96	3.96	31.57	3.27

SD: standard deviation

**Fig 2:**
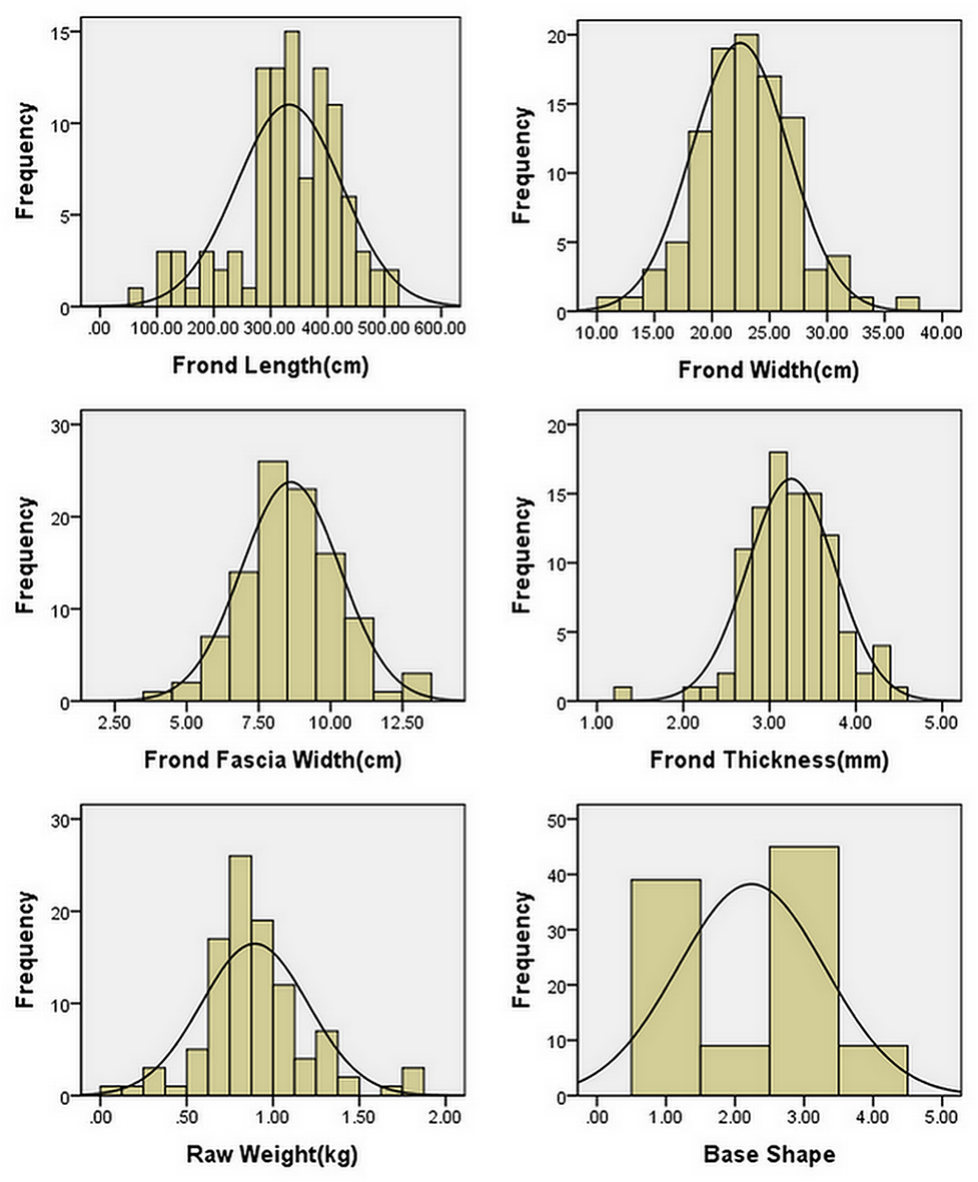
Frequency distribution of the phenotypic value of six traits “Frond Length”, “Frond Width”, “Frond Fascia Width”, “Frond Thickness”, “Raw Weight” and “Base Shape” in F_2_ population.

The statistical parameters for the FL, FW, FFW, FT, RW and BS were examined in F_2_ mapping population. The frond lengths ranged from 67.00 to 513.00 cm, with an average of 332.30±92.40 cm, and the widths ranged from 11.00 to 36.00 cm, with an average of 22.40±4.20 cm, while frond fascia widths ranged from 4.00 to 13.00 cm, with an average of 8.60±1.70 cm. Frond thickness ranged from 1.29 to 4.57 mm, with an average of 3.25±0.51 mm. The raw weight ranged from 0.10 to 1.85 kg, with an average of 0.89±0.31 kg. Values of base shape ranged from 1.00 to 4.00 (evaluation with number according to the angle), with an average of 2.24±1.06. All the data were shown in Table 2. The Kolmogorov—Smirnov test indicated the five traits (FL, FW, FFW, FT, RW) phenotypic variances were coordinate with normal distribution, but “BS” trait fitted discrete distribution (Fig 2). The six traits had great variation, while the absolute values of skewness and kurtosis for these traits were all less than two. These results demonstrated that the studied six economic traits belonged to the typical quantitative traits and they were suitable for QTL mapping analysis.

**Table 2:** Phenotypic performance of the six economic traits in F_2_ family.

Trait	Min	Max	Range	Mean	SD	CV	Skewness	Kurtosis	*P*
FL(cm)	67.00	513.00	446.00	332.30	92.40	27.79	-0.64	0.41	0.25
FW(cm)	11.00	36.00	25.00	22.40	4.20	18.68	0.15	0.80	0.65
FFW(cm)	4.00	13.00	9.00	8.60	1.70	19.73	0.21	0.53	0.06
FT(mm)	1.29	4.57	3.28	3.25	0.51	15.58	-0.20	1.66	0.90
RW(kg)	0.10	1.85	1.75	0.89	0.31	34.54	0.66	1.48	0.14
BS	1.00	4.00	3.00	2.24	1.06	47.48	-0.05	-1.50	0.00

SD: standard deviation; CV: coefficient of variation; *P*: K-S test, *P*>0.05 coordinating with normal distribution

### Segregation analysis

By primer screening, a total of 198 primers with clear, unambiguous polymorphic yielding were applied for genotyping the F_2_ mapping population, including 37 AFLP primer combinations, 22 SRAP primer combinations and 139 SSR primer combinations. 632 polymorphic loci were obtained in total. The dominant markers produced 354 loci (221 AFLP loci and 133 SRAP loci) and co-dominant markers (SSR) produced 278 loci. Of the detected 632 loci, 210 (33.23%) exhibited significant segregation distortion (P<0.05).

For the dominant markers (AFLP and SRAP) analysis, 194 (54.80%) loci followed the 3:1 Mendelian segregation ratio, and 160 (45.20%) exhibited significant segregation distortion (P<0.05). Of the 160 segregation loci, 76 loci embraced bias towards female parent, accounting for 47.17%; 84 biased towards male parent, accounting for 52.83%.

For the SSR study, 228 (82.02%) loci fitted to the Mendelian segregation ratio by the Chi-square goodness-of-fit tests and 50 (17.98%) exhibited significant segregation distortion (P<0.05). Of these 50 segregation loci, 20 loci embraced bias towards female parent, accounting for 40.00%; 30 biased towards male parent, accounting for 60.00%.The information of SSR primers yielding the marker loci mapped on linkage map was present in S1 Appendix.

### Genetic linkage mapping

**Fig 3:**
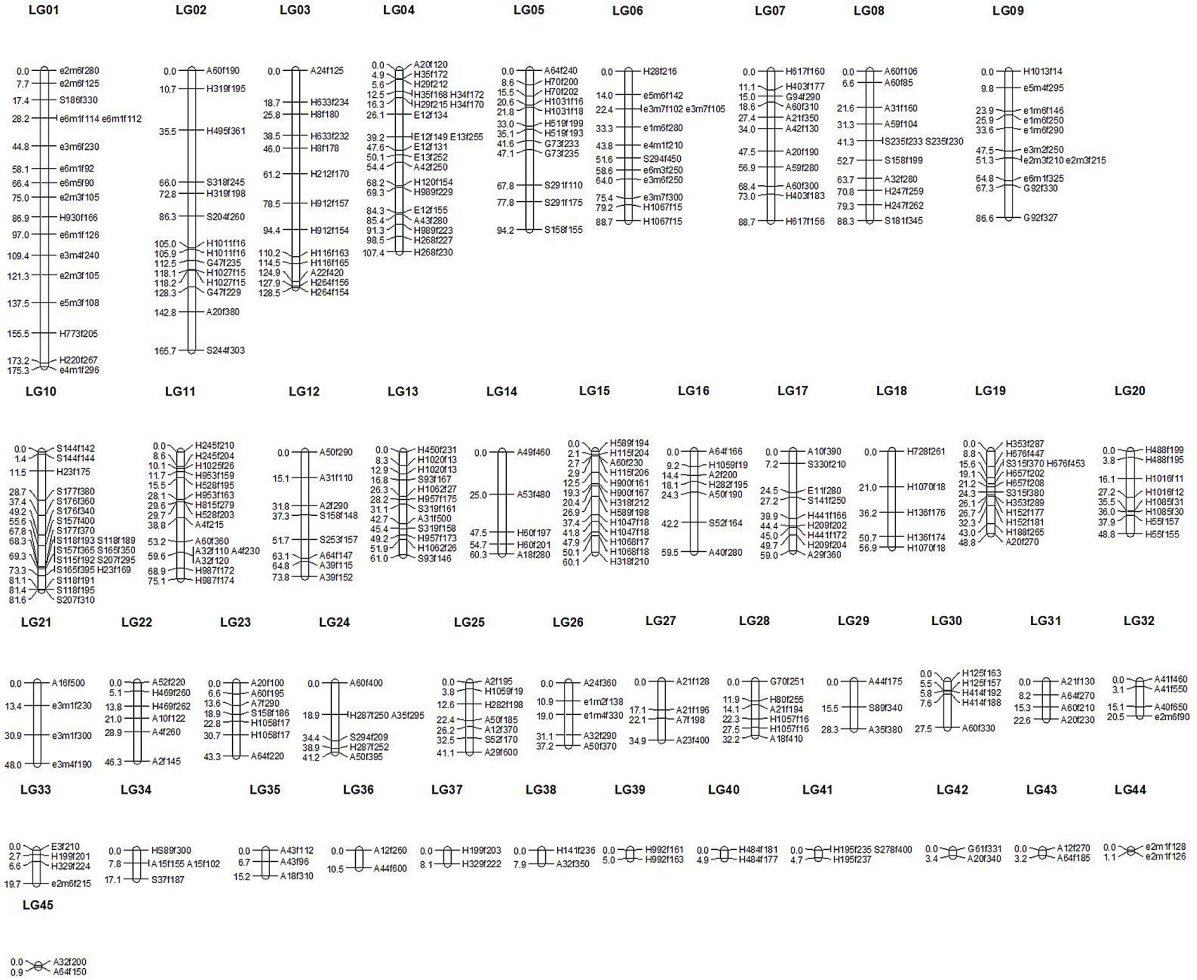
Genetic linkage map constructed by MapMaker software based on AFLP, SRAP and SSR markers. Marker names are shown on the right and the adjacent marker spacing is displayed on the left in Kosambi centimorgans (cM).

A total of 137 AFLP loci, 57 SRAP loci and 228 SSR loci, which fit the Mendelian segregation ratio, were used for genetic mapping construction. Using MapMaker software, of these 422 marker loci, 327 were anchored on 45 LGs, named from LG1 to LG45 (Fig 3). The number of mapped loci on each linkage group was ranged from two to twenty, with an average 7.3 loci per group. The linkage group lengths were from 0.9 to 175.3 cM, with an average of 49.6 cM. The total length was 2233.1 cM, covering the whole estimated *Saccharina* genome (2,186.7 cM) [33]. The yielded map density of the marker loci was 1 per 7.92 cM. Statistical data of the linkage map were summarized in Table 3.

**Table 3:** Summary of genetic linkage maps constructed by MapMaker software.

Parameters the linkage map	MapMaker software
Number of markers mapped	327
Number of linkage groups	45
Average number of markers per group	7.3
Minimum number of markers per group	2
Maximum number of marker per group	20
Average marker spacing (cM)	7.92
Minimum length of linkage group (cM)	0.9
Maximun length of linkage group (cM)	175.3
Average length of linkage group (cM)	49.6
Total map length (cM)	2233.1

### QTL analysis

**Fig 4:**
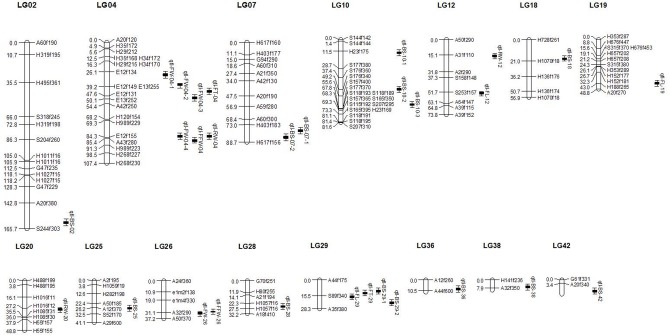
Putative QTLs for six economic traits on the MapMaker linkage map.

Based on the linkage map constructed in this research, a total of 29 QTLs correlated with six objective economic traits were detected on 15 LGs with CIM method. The profiles and characteristics of QTLs were provided in Table 4, Fig 4. Three QTLs correlated with the frond length were anchored on LG12, LG19 and LG29, respectively, accounting for 20.17%, 16.20%, and 2.93% of the phenotypic variance, respectively. The gene actions were all dominant and alleles from QTLs originating from the female strain could promote the length traits expression. Five QTLs correlated with the frond width were anchored on LG4 (four QTLs) and LG26 (one QTL), accounting for 15.56%, 22.11%, 28.42%, 29.05% and 44.91% of the phenotypic variance, respectively. The gene actions were additive, and alleles from QTLs originated from the male strain which could promote the width traits expression. Two QTLs were detected for frond fascia width, which explained 28.18% and 64.00% of phenotypic variance and anchored on LG04 and LG26, respectively. The pattern of two QTLs correlated with the frond fascia width was consistent with the frond width. Two QTLs correlated with the frond thickness, which explained 13.63% and 16.95% of phenotypic variance and anchored on LG04 and LG29, respectively. In addition, the gene actions were all dominant. Alleles from QTLs originated from the female strain which one could promote the thickness trait expression while the other could inhibit trait expression. Of these three QTLs obtained for raw weight, two alleles originated from the male strain which were anchored on LG04 and LG12. One on LG04 promoted traits expression and the other on LG12 inhibited genes about raw weight expression. The third QTL obtained for raw weight was anchored on LG20 and the allele of female parent could promote the increase of raw weight. Fourteen QTLs were obtained for base shape with the phenotypic variance ranging from 1.06% to10.11%. Genetic effects associated with the base shape were quite complex. Female parent allele played a promoting role on base shape at seven QTL loci, while four alleles played an inhibitory role. On the other hand, male parent allele played a promoting role at two QTL loci, while one allele played an inhibitory role.

**Table 4:** Putative 29 QTLs and their genetic effect for six economic traits in *Saccharina*.

Trait	LG	QTL name	Markers interval	Position (CM)	Additive	Dominant	Variance (%)
FL	12	qtl-FL-12	S158f148- A64f147	51.61	-57.0676	66.7719	20.17
FL	19	qtl-FL-19	H152f181- A20f270	42.11	-55.6415	67.6865	16.20
FL	29	qtl-FL-29	A44f175- A35f380	15.51	22.5629	71.7805	2.93
FW	04	qtl-FW-04-1	H34f170- E12f149/ E13f255	27.61	2.6531	-1.1663	15.56
FW	04	qtl-FW-04-2	E12f134- E12f131	36.51	3.3696	-1.6534	22.11
FW	04	qtl-FW-04-4	H989f229- H989f223	82.51	4.1304	-2.7348	28.42
FW	04	qtl-FW-04-3	E12f149/ E13f255-A42f250	48.21	4.2127	-1.7262	29.05
FW	26	qtl-FW-26	e1m4f330- A50f370	30.91	4.6566	3.1389	44.91
FFW	04	qtl-FFW-04	E12f155- H989f223	86.51	1.4498	-0.9683	28.18
FFW	26	qtl-FFW-26	e1m4f330- A50f370	30.01	2.1580	1.2662	64.00
FT	04	qtl-FT-04	E12f149/E13f255- E12f131	42.51	0.2719	-0.3850	13.63
FT	29	qtl-FT-29	A44f175- A35f380	12.01	0.2955	0.5207	16.95
RW	04	qtl-RW-04	H989f229- H989f223	82.51	0.2435	-0.1038	24.53
RW	12	qtl-RW-12	A50f290- A2f290	15.11	-0.1454	-0.0848	9.67
RW	20	qtl-RW-20	H1016f11- H1085f31	27.21	0.1357	0.1416	3.01
BS	02	qtl-BS-02	A20f380-S244f303	159.41	0.4624	-1.9806	9.44
BS	07	qtl-BS-07-2	H403f183- H617f156	83.51	0.4070	0.3993	6.75
BS	07	qtl-BS-07-1	H403f183- H617f156	77.51	0.5049	0.1665	10.11
BS	10	qtl-BS-10-1	S144f144- S177f380	12.01	-0.1602	0.2676	1.06
BS	10	qtl-BS-10-2	S176f360- S157f400	48.01	-0.3674	1.9943	5.26
BS	10	qtl-BS-10-3	S157f400- S177f370	63.01	-0.4669	1.9927	8.16
BS	18	qtl-BS-18	H728f261- H1070f18	18.01	0.1787	2.0139	1.39
BS	25	qtl-BS-25	A50f185- S52f170	26.01	0.3020	-2.0080	4.02
BS	28	qtl-BS-28	H1057f16- A18f410	24.41	-0.2223	1.9783	2.17
BS	29	qtl-BS-29-1	A44f175- S89f340	10.01	-0.2410	-2.0155	2.50
BS	29	qtl-BS-29-2	S89f340- A35f380	21.51	-0.2517	-2.0124	2.69
BS	36	qtl-BS-36	A12f260- A44f600	8.01	-0.3621	1.9721	5.34
BS	38	qtl-BS-38	H141f236- A32f350	6.01	-0.3008	1.9964	3.88
BS	42	qtl-BS-42	G61f331- A20f340	10.01	-0.2272	0.0343	2.00

## Discussion

### Mapping populations

Selection of parents in hybridization was important for genetic linkage mapping. If the parents were close in phylogenetic relationship, the proportion of polymorphism loci from the hybrid progenies would be too low to construct high-density genetic linkage maps. If the relationship was too far, pairing and recombination were suppressed during the process of meiosis, which resulted in the low recombination rate, could lead to serious distorted segregation and even make the hybrid sterility. While *Saccharina* hybridization among different species would not lead to sterility and inbreeding depression [39]. Therefore, we selected *S*. *longissima* and *S*. *japonica* with distant relationship, as parents for hybridization. Obvious morphological and genetic differences between the two *Saccharina* species [40] made them suitable as hybridization materials for high density genetic linkage mapping and easy to separate quantitative loci.

Doubled Haploids (DH), Recombination Inbred Lines (RIL) and F_2_ populations were commonly used as biparental genetic populations in QTL linkage mapping [41]. The length of time needed for producing RIL populations was the major disadvantage, because usually six to eight generations were required. However, the production of DH populations was only possible in species that were amenable to tissue culture (e.g. cereal species such as rice, barley and wheat). F_2_ populations, derived from F_1_ hybrids, have been regarded as the simplest type of mapping populations developed for self pollinating species [42]. *Saccharina* was hermaphrodite and had no reproductive isolation. So we chose F_2_ population for genetic map construction and QTL detection in this study. In the F_2_ population, all the genotypes from parents existed, therefore, additive effect and dominant effect between genes could be detected. In other words, more genetic information about the whole genome could be obtained from the F_2_.

The size of mapping population was another important factor affecting the density of genetic linkage map and accuracy of QTL detection [43]. Population sizes used in preliminary genetic mapping studies generally ranged from 50 to 250 individuals [11]. Generally, the large mapping populations could yield more accurate results. The genetic linkage map of *S*. *japonica* and *S*. *longissima* was constructed by Li using 60 sporophytes whose genome coverage was very low [17], and a tentative AFLP-SSR linkage map of *Saccharina* was constructed using a haploid population of 40 gametophyte clones isolated from an individual of Dongfang No.2, covering 66% of *Saccharina* genome [20]. When F_2_ mapping population with 92 individuals was used to construct a *S*. *japonica* genetic map, the map was up to 1,811.1 cM covering 82.8% of the estimated genome [19]. With 102 F_2_ hybrids in this study, the total length of 2233.1 cM covered the whole *Saccharina* genome. Taking into account the time and costs, this experiment showed that the mapping population which reached about 100 individuals could meet the requirements of “framework” genetic linkage map construction.

### Genetic DNA markers analysis

Over the past few years, a number of PCR based marker technologies such as RAPD (Random Amplified Polymorphic DNA), AFLP and SSR have been applied to genetic linkage map. Choosing different types of markers, dominant markers or co-dominant markers, would influence on the precision and accuracy of genetic linkage mapping. The use of dominant markers to construct maps tended to produce larger gap, but also easy to appear smaller fragments of linkage group, like diad, triplet. SSR markers that were abundance, high level of polymorphism, high reliability and detect co-dominant multi-allelic loci could identify the homozygous and heterozygous loci [44]. They would be conducive to further integration of different linkage groups. In plants, SSR markers have been successfully used to genetic linkage map construction such as peanut [45, 46], cassava [47], *Cucurbita moschata* [48], *Brachypodium distachyon* [49]. However, the previous genetic linkage map of *Saccharina* mainly focused on AFLP markers and only a few SSR markers were available [17–20] because developing SSR involved time-consuming and labor-intensive [50]. For the construction of a high-density genetic linkage map, we have constructed the *Saccharina* SSR enriched genomic library and developed SSR markers on a large scale [30]. In this work, 37 AFLP markers with 221 polymorphic loci, 22 SRAP markers with 133 polymorphic loci and 139 SSR markers with 278 polymorphic loci were screened. Newly developed SSR markers were mapped in intervals of fragments with AFLPs and SRAPs in order to decrease the map distance between markers. To our knowledge, this was the first genetic linkage map of *Saccharina* with SSR markers as the main marker type as well as SRAP markers introduced firstly for genetic linkage mapping. At the same time, this genetic linkage map employed the most DNA markers during all the *Saccharina* genetic maps so far.

In addition, genetic linkage groups obtained in this study were anchored by one or more SSR markers. For more than two SSR loci on the same linkage group, the arrangement of SSR amplification fragments on *Saccharina* chromosome was deduced according to SSR loci order on the linkage group. If the position of two SSR loci on linkage group was close enough and the SSR amplification sequences were known, sequences could be assembled. Given that the SSR markers will serve as anchor points in comparative mapping and integration different linkage maps, these studies will facilitate the integration with physical map and lay the foundation for sequence map of *Saccharina*.

### Segregation distortion analysis

Segregation distortion was prevalent in many plant species [51]. Significant frequencies of segregation distortion have been reported in several species, including 41% in *Cryptomeria japonica* [52], 30% in coffee [53] and so on. For previous genetic maps of *Saccharina*, 30.6% reported by Li et al. [17] and 33.3% reported by Liu et al. [19] exhibited significant segregation distortion. In this study, it showed 210 segregation distortion sites during F_2_ populations, and the segregation distortion frequency was 33.23%. These results showed that segregation distortion frequency obtained in this work was consistent with existing *Saccharina* reports. Segregation distortion was closely related to the type of genetic populations. Although, comparisons have shown that segregation distortion was more prevalent in DH and RIL than in F_2_ populations [41], many researches indicated that the segregation distortion ratio in interspecific population was higher than that in intraspecific population [54, 55]. Thus, the relatively distant genetic relationship between female parent (*S*. *longissima*) and male parent (*S*. *japonica*) would contribute to the observed high frequency of segregation distortion in this study. The estimates of recombination frequency between co-dominant markers were thought to be less biased by segregation distortion than estimates of recombination between dominant markers [56]. Here in our analysis, 84 (38.01%) AFLP marker loci, 76 (57.14%) SRAP marker loci and 50 (17.98%) SSR marker loci showed segregation distortion (P<0.05). These results proved that dominant markers provided poor information in the case of segregation distortion and, therefore, co-dominant markers should be used as many as possible for construction of the linkage map in the future.

Although segregation distortion could affect the order of markers on a linkage map as well as distances between markers, most studies indicated that removal of segregation distortion would result in loss of large amount of information, reducing genome coverage and thus leading to missed important QTL sites [57]. However, we excluded the distorted loci in genetic mapping because it might decrease the accuracy of the map distances and QTL detection. Segregation distortion has been increasingly recognized as a potentially powerful evolutionary force and would affect the construction of genetic linkage map [51]. Doucleff et al. suggested that markers deviated from the expected segregation ratio at the 5% level but not at the 1% level should be included in mapping procedures to reduce the frequency of false positives [58]. This approach will increase the marker coverage of the genome [59], which we should pay more attention in the coming research.

### Genetic linkage maps

Commonly used software programs included MapMaker/EXP and MapManager QTX, which were free on the internet, while JoinMap was another commonly-used program for constructing linkage maps [60]. In addition to MapMaker software, JionMap was also attempted to use in our research in order to avoid the interference with the mapping results by different mapping softwares under the condition of the same mapping populations and the same molecular markers. By comparing the results of MapMaker and JionMap in total map length, number of mapped markers, average marker spacing, average length of linkage group and so on, the genetic linkage map with higher density made by MapMaker software was used for the subsequent analysis and application. Similar results were obtained with *Acacia mangium* [61] and *Picea glauca* [62]. This result might be due to different site ranking methods during the mapping processes by two kinds of software.

The current map was totally 2233.1 cM, covering the whole *Saccharina* genome, with an average marker distance of 7.92 cM. Compared with the previous map of *Saccharina*, the genetic map presented in this research was greatly improved. The suitable density and the high coverage of the map offered favorable condition for QTL analysis.

45 LGs were obtained which consisted with the number in the *Saccharina* map reported by Yang [20] and more than other previously reported by Li (14 LGs) [17] and Liu (28 LGs) [19]. The chromosome number of *Saccharina* haploid has not yet been determined confidently, ranging from 16 to 32 reported by different researchers [63–67]. LGs (45) obtained in the map developed in this research were higher than the chromosome number, and a one-to-one correspondence between the linkage groups and the chromosomes was not determined. This could be attributed to several factors such as bias in collection of *Saccharina* used for mapping population, types of markers used on map construction, as well as density and distribution of markers. In order to resolve this problem, some markers on each linkage group could be selected in situ hybridization to determine its position in the chromosome. Alternately, genetic linkage map was re-constructed using the same molecular markers but different mapping populations and the same linkage group from the two maps was likely to represent one chromosome.

### QTL analysis

Genetic linkage maps allowed a complete identification and the location of QTLs for MAS. Based on phenotypic data of six economic traits, a total of 29 QTL sites were identified in this work including three QTLs for FL, five QTLs for FW, two QTLs for FFW, two QTLs for FT, three QTLs for RW and fourteen QTLs for BS.

For FL trait, alleles from QTLs all originated from *S*. *longissima* with long frond and the gene actions were all dominant, while for FW and FFW traits, alleles from QTLs all originated from *S*. *japonica* with wide frond and showed all positive additive effect values. Thus, it can be expected that FL, FW and FFW traits could be well inherited in progeny from parent with obvious excellent characters. For FT trait, two QTLs both originated from *S*. *longissima*, while one allele played an effective role and the other played an inhibitory role. It indicated that genes had interaction, but the mode of interaction for this trait remained to be studied further. For RW and BS traits, the results in this work showed that the regulation mechanism was more complex: female parent and male parent played a promoting role in traits expression as well as an inhibitory role. Taking into account the changing morphological characteristics of base shape during different growth periods, BS trait of *Saccharina* was a complex quantitative trait, which was controlled by several genes. Identification of QTLs influencing several traits could increase the efficiency of MAS and enhance genetic progress [68].

Of these, some QTLs controlling different traits were distributed in clusters, especially on LG 04 where three QTLs controlling frond width, frond fascia width and raw weight respectively were clustered in one region and four QTLs controlling frond width and frond thickness clustered in another region. In QTL mapping of crops, this phenomenon was often detected [69, 70]. The above findings may be attributed to the following: (1) Genes controlling different traits were tightly linked, thus, they were located either within the same region or in adjacent locations on the same chromosome; (2) a single functional gene might play a regulatory role for a series of genes; (3) a single gene could independently control either two or more different traits [71] and (4) the density of genetic linkage map was not enough to separate QTLs controlling different traits in one linkage group. However, limitations of mapping efforts along with experimental errors, which were difficult to avoid, may also contribute to positioning QTLs without linkage relationships to be localized within the same or adjacent regions of the linkage map. Thus, authenticity of a QTL must be verified by comparing QTL results from different environments or different mapping populations [72].

In conclusion, in this study, SSR markers along with AFLPs and SRAPs were used for constructing linkage maps of *Saccharina* which provided an effective tool for genetic analysis. Furthermore, the marker distance on this framework map and the good coverage provided enough marker density for identifying quantitative traits. A total of 29 QTLs for frond length, frond width, frond fascia width, frond thickness, raw weight and base shape have been identified. All of these findings will be useful for subsequent studies, including gene tagging efforts, MAS breeding, and comparative genomic studies. Furthermore, the whole genome sequencing of *S*. *japonica* has been recently completed (unpublished data), and thus, this genome sequences along with findings obtained in this study would greatly advance efforts in characterizing the *Saccharina* genome and identifying genes associated with quality traits. Further research of quality traits will help us to breed high-quality variety.

## Supporting Information

S1 Appendix Characteristics of the polymorphic microsatellite loci of *Saccharina japonica*. (DOC)
