# Supplementary material for: Genetic Map Construction and Quantitative Trait Locus (QTL) Detection of Six Economic Traits Using an F2 Population of the Hybrid from Saccharina longissima and Saccharina japonica
Source: PLoS One. 2015 May 26;10(5):e0128588. doi: 10.1371/journal.pone.0128588 (PMC4444332; doi:10.1371/journal.pone.0128588)
Supplement: S1 Appendix — (DOC) [file pone.0128588.s001.doc]

**S1 Appendix. Characteristics of the polymorphic microsatellite loci of *Saccharina japonica*.**

| **Primer Name** | **Core Sequence** | **Forward Primer(5'- 3')** | **Reverse Primer(5'- 3')** | **Product Size(bp)** | **Ta (℃)** |
| --- | --- | --- | --- | --- | --- |
| ESTSSR003 | (GCA)6 | TTTGGGGACAAACTACTTGG | AACTAATGGTTCGTAATGGTG | 201 | 51.7 |
| ESTSSR011 | (AC)9 | TACTGCACTGAGTGTAGTATCTC | CGACGACGCCAAATGTAATG | 259 | 50.8 |
| ESTSSR012 | (TGC)13 | GACGCTGCAAACCGACTTCT | GCTGGACCTTCACGTACCTTG | 239 | 55.7 |
| ESTSSR013 | (TGC)13 | TTCTTGTTTCAGTGCCAGTTC | CGCAGCTTTACCCTCCTCTT | 252 | 55.1 |
| HDGS016 | (AT)7 | GGAACAGTTTGCTGCTTT | TGGAGTCAGGCTCTTCTT | 254 | 47.3 |
| HDGS023 | (AT)5 | ATTATGGCAGGTGAGGAA | CAGGTGCAAAGTTCTAACAG | 396 | 48.3 |
| HDGS047 | (GCC)8 | CTCGGCTTATTGTTTGCT | TGCTTTACATCGGAGTTTT | 235 | 50.0 |
| HDGS061 | (TC)11 | GAACGGTTGCCCATCTCC | TTCGCCTGCTCTTCTTGC | 331 | 51.6 |
| HDGS070 | (GA)9…(GA)6 | CTTGCCGAGTAGATGGAGA | TACAGCGAGAACCCGAAC | 251 | 52.4 |
| HDGS073 | (TGC)9 | CCGATGTTGGGTTGACTG | ACAGCAGGTGAGGGTATTTT | 235 | 52.7 |
| HDGS092 | (GGA)5 | AAGGAGCGTGGAGAACTG | TACTCGTCTTGTGGTGGG | 327 | 55.1 |
| HDGS094 | (ACA)6 | AGCCTGTGACCACGGGAATC | CCAAACGCCCAAACACCA | 293 | 55.9 |
| HDS0008 | (CG)5 | GAGTTAGGCACCCGTTTTCT | GCGGGTTGCTGTCAGTTT | 180 | 52.2 |
| HDS0023 | (GT)7 | TCACAGAAAATGAGGGGAGG | GCACAAGAAACCATACGAGG | 175 | 52.7 |
| HDS0028 | (CA)5 | AAGCAGCCAGCCAACAAC | TGGAATCGCTTGGCACATAT | 214 | 52.7 |
| HDS0029 | (GCA)6 | GCAGTGTATGCGAAACGAAG | TACGACGGCAACCGATAAC | 215 | 52.7 |
| HDS0034 | (AG)11 | AAAGCCTCCTCACTATACCAC | GCTAAGAACGCACGCAAG | 170 | 53.0 |
| HDS0035 | (AG)12 | AAAGCCTCCTCACTATACCAC | GCTAAGAACGCACGCAAG | 172 | 53.0 |
| HDS0039 | (AT)6 | ACGGAATGGAGGAGTGGCT | ACCTGGTATGCGTGCTTTT | 257 | 53 |
| HDS0044 | (GA)5 | CATCGCTCTGGGGATTATTT | TTTTGTCACGACTGGCTGAT | 170 | 53.2 |
| HDS0055 | (CAG)5 | CCGTTGTCCACTGCATGAT | GCAGCACCGCTTAACTCCA | 157 | 53.5 |
| HDS0060 | (ATGC)5 | TACCGCACTACAAGGGAGC | GAGGCCAACATCGTCAACT | 201 | 53.5 |
| HDS0070 | (CTA)6 | TCTATCCAAGATTGCACGAT | ACATGAACGACGAGCTAAAC | 202 | 53.7 |
| HDS0080 | (TGC)5 | GCAGTCGGTAGGGTTGTGAG | TGCTAAGCCAGCCAATCAAC | 252 | 54.1 |
| HDS0087 | (GT)5 | AGGCAGCAGGCGAAAGACAT | GGGCATTGGCGATAAGCAAC | 195 | 54.3 |
| HDS0092 | (AC)5 | GCGTCGTGCGATACAAGTG | TTTGTTTGTCGGGGCTTT | 190 | 54.6 |
| HDS0102 | (CT)6 | CGCAAGGATAGCATGTCAGT | GGGATGTAGGGGCGTTAGAT | 237 | 55.6 |
| HDS0115 | (TG)5 | GCTCTGTTTACCGATTGCTC | TGTCCTGTCGTGTATCCCTC | 214 | 49.6 |
| HDS0116 | (GT)5 | CAATCCGCCATAAAGTCGT | TCATCAGAGGCTTCATCCAA | 165 | 49.7 |
| HDS0118 | (TA)5 | TTCGTTTTGCTTCGCTTTAG | CCATAACTCGTCTCGGGGTC | 206 | 49.8 |
| HDS0120 | (TA)7 | CGGTAGCGTCTTTTGTTTTC | ACCTGGTGTATGCTGTTTGC | 154 | 50.0 |
| HDS0125 | (TA)6 | ATAGTTGTCGGACATCTCAGG | CGGACGCTTCGTACTTGTT | 157 | 50.3 |
| HDS0125 | (TA)6 | ATAGTTGTCGGACATCTCAGG | CGGACGCTTCGTACTTGTT | 157 | 50.3 |
| HDS0136 | (CA)6 | CGCACAACGCCTCATAAAT | TGCCGAGGAGTTGCTATCT | 176 | 50.7 |
| HDS0141 | (TCT)6 | TTTTCGGGAATATCGGTCTC | AGGGCAAGTCAGTTCATCG | 236 | 50.9 |
| HDS0152 | (AT)8 | GCCTGGCACATGGAAATAC | CTCACTCAGCAGAGCAGACAC | 177 | 51.1 |
| HDS0167 | (CT)9 | GCGTATAAATAGTCCAACCCA | TACTTCGTGCCGCTCTGA | 191 | 51.3 |
| HDS0175 | (TG)5 | GTGCGTTTTGTTTGGTTTCG | GCGTGCTATAACAGCGTGAA | 158 | 51.4 |
| HDS0188 | (GT)5 | CTGTTTATTAGCAGGTGGC | TGGACTTTCGTGGAGTTTCA | 265 | 51.7 |
| HDS0195 | (TC)5 | CTGTTCCTCTGCTTATGCCC | CACGGTAGCGATATGGTTGAT | 235 | 51.8 |
| HDS0199 | (CAC)5 | CAACCCACCCAACCAACT | CATCTCGTAACGGCAGCTATA | 201 | 51.8 |
| HDS0203 | (GT)5 | AAACCGTCCTTTGTACGAGTG | GCATTGTCTGCTCTGCTTACTG | 153 | 51.9 |
| HDS0209 | (TAC)8 | CCGCAGATCGAAAGGAAAC | CAACCAGCCATCACCCAT | 204 | 52.0 |
| HDS0212 | (TC)6 | GACAGTGGCGAAGAACAAAG | CAGAACACTCAAAACCCATACA | 170 | 52.1 |
| HDS0220 | (GA)7 | TTGCTTCGTTGATCCTTCAC | GGAGATTTTCATTCCCGTTT | 267 | 52.2 |
| HDS0245 | (AGT)5 | CAAGCAAGCGACGGTCTATC | TGGTGTATGGGATGGGATT | 210 | 52.6 |
| HDS0247 | (GAT)8 | CAGCATCCTCATAACGACTACG | GGGGTACATTTTGAGCGTC | 262 | 52.6 |
| HDS0264 | (GT)5 | AAGAATCAGGTCTGGGCACT | CGCATCTCACTTTAAGGAGCA | 154 | 52.9 |
| HDS0268 | (GCT)6 | CATTCAAGACCCTTGCTCG | TCGTGGTGTCGCTTTCTG | 227 | 53.0 |
| HDS0282 | (ATG)6 | AGGCAGAGGCACGAGCAGAAC | CCCTAACGACCGAATGGAA | 198 | 53.3 |
| HDS0287 | (GT)6 | AGACCACTCGCTTTATTTTGAC | TGCGTACCGACCACAGACA | 252 | 53.4 |
| HDS0294 | (CA)5 | ATTATGAGCACGACAGACGC | GGTTGGAGTGGTCACGAAA | 177 | 53.5 |
| HDS0307 | (GA)7 | TCGTGTTCATCGTCTTATTCTG | TTGCGTCTTGCTTACTCCC | 280 | 53.7 |
| HDS0318 | (GCT)6 | GTAGGATAGCCCCATTTGCC | TGCTGTTGCCACGTTCATT | 210 | 53.8 |
| HDS0319 | (CTC)7 | GAACCCCAACGACTACGAG | TCCCTGAGGTGCCCTATCT | 195 | 53.9 |
| HDS0329 | (GCA)5 | CCGATTGTCTGATTGAGGACT | TAGAGGATGTGCCCAGAGTG | 224 | 54.0 |
| HDS0337 | (TC)5 | AAGCAACACGCTGGAGGT | CAACAAGGAGGGGAGTAAGG | 218 | 54.1 |
| HDS0353 | (ACT)7 | GACCGAATGTCAAGCCAACC | AGTCCCAGTCGCCAGAAGTG | 287 | 54.4 |
| HDS0403 | (CTA)12 | TTCTTGGGGTCACATCGTC | CCGTAAAGTATTTTGCGGTAGT | 177 | 50.0 |
| HDS0414 | (TA)6 | TCGGACATCTCAGGAAGG | AACTGGCGGCATTATTTT | 188 | 51.0 |
| HDS0441 | (AG)5 | GGGTTGTTGGTGGGAGATT | GGACGGCGTTTATACCTACTGT | 166 | 51.9 |
| HDS0450 | (AAG)5 | GAAGGAAGGGGTGGAAGAC | ATCATGCAGGTTATGCGACT | 231 | 52.1 |
| HDS0469 | (ATC)7 | AAACAAAACGCCTTGCTCC | GAAACGACGATGACAAATGG | 260 | 52.4 |
| HDS0484 | (AG)5 | TGTCATCAGTGGCATCAGTTT | GGTCACAGGGATAGTTGGAAG | 181 | 52.9 |
| HDS0488 | (GCA)5 | CCTCCCAGCGTTACAGAT | GTCCGACAACCAACAAGC | 195 | 52.9 |
| HDS0495 | (GTA)10 | GCCATTGAAACCCGACAC | TCCCACAAAGATGCAAAACT | 357 | 53.1 |
| HDS504 | (ACT)5 | ACCCACGACCGTTGATTC | CCATGTTTTCGATAGGAGTTG | 357 | 53.3 |
| HDS0519 | (AGC)5 | GCGGAGTCAGCGAGATGTT | CGGTCGTTACTGGTATGGG | 199 | 53.6 |
| HDS0528 | (GCTT)8 | CTCAAGGACGCTGTAAGAAG | AAGGGAAGGAGGGATGTT | 203 | 53.8 |
| HDS0589 | (TAG)7 | ACACCCTAAATCCAGTTCAATC | TGCGGCTTCCCTATCACA | 196 | 49.8 |
| HDS0593 | (ACT)6 | TCACGGAAGGCAAGAAACT | CTCGCCTTTTAATTGTGGTT | 157 | 50.0 |
| HDS0608 | (ACAG)6 | TGGTGTTTACACGCTACGATG | TGCTAAGTTTTGCTTCTCCCT | 209 | 50.9 |
| HDS0617 | (AC)6 | AGCAGAGGCCGAAGCAAAT | CGTGGGACATATCGCAAAT | 156 | 51.3 |
| HDS0633 | (CT)6 | ATTGCTTGGCAGTTTAGAGTTG | ACGCACAGAAGACGAGGGT | 234 | 52.0 |
| HDS0642 | (ACT)6 | CTTCCACCTCCACCACAAC | TTACCCAATTCCGCAACAT | 237 | 52.3 |
| HDS0657 | (GA)5 | ATTTGGACGCTTTGCTGTT | TGCTGCTTTACTTTTGACCC | 208 | 52.6 |
| HDS0671 | (TC)5 | ATGGTATCTCATCGTCTTCGTT | GTCACTTGGTCCGTTCTTTG | 210 | 53 |
| HDS0676 | (CG)5 | CAAGCACGAGTGATAAAGGG | GTGGTCAGGTAGAACAGAAAGG | 447 | 53.1 |
| HDS0728 | (TG)5 | CAGAAATGGACGCCAAACG | CACGACATCGCAACAAAGC | 261 | 53.8 |
| HDS0756 | (ACAAA)5 | ACCTACGTCACCGCATCAT | GAGCGACAGTGCCTTTTGA | 257 | 54.4 |
| HDS0773 | (CT)7 | CTCCCTTCTTTCCCTTCG | GGACCATCGGCTTTCGTT | 205 | 55.0 |
| HDS0900 | (TC)6 | TCGCAAGCAGACACCAATC | GGGTCTGAGGTACGACGATTA | 167 | 51.7 |
| HDS0906 | (GTAT)5 | GGGGCTCCATAAGTGAGAAG | CATACCACAGGTAATGCCTACA | 157 | 51.8 |
| HDS0912 | (GAT)9 | GACATCCGGCTTTCGTTC | TCGCCATAATCATCCTTGC | 157 | 52.1 |
| HDS0919 | (AGT)6 | CGCCTTTTAATTGTGGTTCC | CCTAGAATGTTTGCCGTGAAG | 214 | 52.4 |
| HDS0930 | (AG)5 | CGTCGGGTGGGGTAGATTT | CCGTGCAGTCACTACGAGGT | 164 | 53.0 |
| HDS0953 | (CAACC)5 | GAAGTGGAAAACGACGTATG | ATCGATCTAGCCGGAGGGT | 159 | 53.9 |
| HDS0957 | (GCA)6 | CACTCAATGTCTTCGTCAATCG | GTGCAGTGGCTGACAGTGTAG | 173 | 54.1 |
| HDS0987 | (TA)6 | CCGCCATCACCACCATCTAC | GCCGTGTTCACGGGGTATAT | 172 | 55.8 |
| HDS0989 | (AC)7 | AACAGCAGTGCCCCGACCAA | AAGGCTTTCCGCTTGCGAC | 223 | 55.9 |
| HDS0992 | (GT)5 | TTACCGCCCAGACGCCCTACTA | ACCGAAACCACCAGGCTCCA | 161 | 57.3 |
| HDS0994 | (GC)5 | GAGCGGACAGACCCAGATGT | TTCGAGGCCCTGAAATCC | 249 | 57.9 |
| HDS1011 | (ATC)6 | CGGCACAGCAGACAAGAC | ATGAGCGATGATAGCGACT | 164 | 49.0 |
| HDS1013 | (AT)5 | CACATCGCACGGGACATA | AAACCTCAATCGCACCAG | 148 | 49.0 |
| HDS1016 | (GT)6 | CGTTGTCCTTTAGATGCT | CAGGAGCCAAAGAGTATG | 119 | 49.1 |
| HDS1020 | (TTGA)7 | GTGATTGTTATGGTTTATTGCTTG | TCGACGAATGGGGACATG | 133 | 49.5 |
| HDS1025 | (AGT)5 | CCTCTGCTTATCGTTTTC | TAGCCACTATTGTAGCTCAT | 269 | 50.0 |
| HDS1027 | (GA)9 | AATACAGGCAAAGAGCAAC | CCTTGAAGTTCGGAGGAC | 152 | 50.3 |
| HDS1031 | (CT)8 | GTGCTACCCTCACCATTG | TTCGTTGACGATAACAAACT | 169 | 50.4 |
| HDS1047 | (GT)5 | TCTTCGTCCGTTCTTGTCT | ATATGATACCGGCGTTGTT | 183 | 51.2 |
| HDS1057 | (GA)5 | TGTGGCAGCTTGTTTCGT | CGTGGTTACCGTATGTATTTG | 162 | 51.7 |
| HDS1058 | (ATCC)7 | AATACACCCGACCGAAGC | CAGGCAGACTAGCGCAGA | 174 | 51.7 |
| HDS1059 | (ATG)6 | CAGAGGCACGAGCAGAAC | CCTAACGACCGAATGGAA | 194 | 51.9 |
| HDS1062 | (CTG)5...(TA)7 | GCATCATCATTCGGCTTCT | TGGGTAAACCGCTATTCG | 268 | 51.9 |
| HDS1067 | (GC)5...(GT)6 | TGCGTCCTCGTACTACTTTAGC | AGGAAACTTCTAGTGGGCAAA | 152 | 52.1 |
| HDS1068 | (TC)5 | GTGGTCACAGGGATAGTTGG | TCATCAGTGGCATCAGTTTT | 181 | 52.1 |
| HDS1070 | (AGC)6 | AAAAGGGACGAAGCACAG | AAACGGGTAGATACATACGAT | 187 | 52.1 |
| HDS1085 | (GTCGG)5 | TGGCACAGGTCCAACTCA | AGGTCGCCCTTACCCAAC | 308 | 52.7 |
| SSR037 | (GTGA)4 | GGAAACGGGCAAACCATA | TCACCCAAATACCCACCC | 183 | 53.5 |
| SSR052 | (CAG)6 | TTTGAGGCTTCAACGCTAT | TTGTGAATGCTTCCTCCTG | 166 | 51.8 |
| SSR089 | (GA)15 | GCGGGCTTGCTTGAAACG | GCCAGGCGGTCTGACGAT | 347 | 57.9 |
| SSR093 | (TGC)6 | GACGGGTGTACGATGTGC | AACTTTCTAAAGCGAACGATG | 167 | 54.0 |
| SSR115 | (AG)47 | CGGCGCACGTAATTCTCG | AGCCTGGGACTGCTCCCTCA | 219 | 55.4 |
| SSR118 | (TG)7 | AGACAGGCGTTGGCGTAG | GAGCGAACATCAAACCATTAC | 193 | 53.9 |
| SSR141 | (CAT)5 | GGGTGATGACCTTGGATG | ATGAGGTTCCTGGGCTGT | 252 | 52.1 |
| SSR144 | (GT)9 | GTGTAGTCTATCTATGCGTGTC | CATTAGTGAGCCCATGTCT | 280 | 50.8 |
| SSR149 | (TCGG)9 | GGAATACTCGTATCGCTTAG | CAGACCGACAAACAGACC | 388 | 50.2 |
| SSR157 | (GT)5 | GTACAGGAAGAGCGAGCCT | TCTTTCACTTCACTTCAGGGT | 350 | 51.1 |
| SSR158 | (TGC)5 | CACTGAGCTTTACATGGGA | TTATCGTCGGGTGCTACT | 176 | 54.3 |
| SSR165 | (GT)5 | GTGCGTACAGGAAGAGCG | TCACTTCAGGGTCATTTCAC | 346 | 50.1 |
| SSR176 | (TG)6 | TGTAGATTTGAGTATCGTGGGT | TTTCATTGTTGCGTTTCG | 341 | 51.3 |
| SSR177 | (GT)5 | GTGCGTACAGGAAGAGCG | ACTTCACTTCAGGGTCATTTC | 350 | 51.0 |
| SSR181 | (TC)12 | TGGCAACACCAAGAATACG | ACAAGCCGAGATGAAACG | 345 | 53.9 |
| SSR186 | (CA)7 | TTTCATTGTTGCGTTTCG | AGATTTGAGTATCGTGGGTGT | 338 | 51.3 |
| SSR204 | (GA)24 | GGCATGTAGAAATTCAAGGG | GCTGCTAATAGTCACGAGGG | 260 | 55.4 |
| SSR207 | (GT)5 | GTGCGTACAGGAAGAGC | GACAAATATCGCACCACA | 289 | 52.0 |
| SSR217 | (GA)5 | TCGGTACGAAGGGTGAAA | ACGCCACTCGTCAAATGC | 317 | 53.4 |
| SSR235 | (GCT)7 | CTCTGCTGGCTGCTACTT | CTGGCTTGCGTCGTGATA | 230 | 53.3 |
| SSR244 | (TC)23 | CTTCGGTACTGCCATCGG | GAGGGTGCTTTATACACTATTT | 299 | 52.9 |
| SSR253 | (ACA)9 | AGCGTGTAGAAGGGAGCG | GCACTAAAGCCCGTAGCC | 328 | 53.4 |
| SSR275 | (AC)5 | AATGGTTGGTGCTGTTCT | CTTATCATCATACGACTACGG | 259 | 53.0 |
| SSR276 | (TC)26 | ATACGAGCAGCGTGAGCC | CAAGCAGGTGGGTGGAGA | 191 | 53.8 |
| SSR278 | (AG)23 | TCCCATATTAGTGGTCAAA | TAGAAGCGTCAATCGGTA | 399 | 50.4 |
| SSR291 | (CACCAA)4 | CGACACCATCACCCACTA | TTGAACGACTCGGACAGC | 208 | 51.5 |
| SSR294 | (AATAA)4 | CAAACCAGGAACCGTAGTG | GAGTAGACCCACCTTATCAC | 209 | 48.6 |
| SSR315 | (ACAAC)5(ACAAA)6 | AAAGGTCGATAAGCTCGCAGTT | GCGTTGTTTCGCAAAGTGATT | 379 | 59.0 |
| SSR318 | (AC)9 | AAAAGGTGAAGGGATTCGTCG | CTGAGCAGATTTATGTGGAGCG | 241 | 59.0 |
| SSR319 | (AC)11 | CGTGGTAGAACTGCGTAGCG | AGTGACATCGTTGCCCGTAT | 161 | 51.0 |
| SSR330 | (CAG)5 | AACCGCTCTTTCCACCCA | TCAAGGCAAGGACCGTAG | 290 | 56.0 |

Ta: the optimal annealing temperature
